# Prevalence of *Yersinia *Species in Traditional and Commercial Dairy Products in Isfahan Province, Iran

**DOI:** 10.5812/jjm.9249

**Published:** 2014-04-01

**Authors:** Ebrahim Rahimi, Sara Sepehri, Farhad Safarpoor Dehkordi, Shima Shaygan, Hassan Momtaz

**Affiliations:** 1 Department of Food Hygiene and Public Health, College of Veterinary Medicine, Shahrekord Branch, Islamic Azad University, Shahrekord, IR Iran; 2 Faculty of Agriculture, Shahrekord Branch, Islamic Azad University, Shahrekord, IR Iran; 3 Young Researchers and Elites Club, Shahrekord Branch, Islamic Azad University, Shahrekord, IR Iran; 4 Department of Microbiology, College of Veterinary Medicine, Shahrekord Branch, Islamic Azad University, Shahrekord, IR Iran

**Keywords:** *Yersinia enterocolitica*, Milk, Dairy Products, Seasons

## Abstract

**Background:**

*Yersinia* species, especially *Yersinia enterocolitica,* are considered as the most prevalent milk-borne pathogens. Several serological and molecular techniques have been developed for rapid and safe diagnosis of yersiniosis.

**Objectives:**

This study was carried out to assess the prevalence rate of *Yersinia* species, especially *Y. enterocolitica,* in milk and dairy products in Isfahan province, Iran.

**Materials and Methods:**

A total of 285 commercial and traditional dairy products as well as 267 pasteurized and raw milk samples were collected during one year. The samples were studied by culturing and the positive-culture samples were investigated using PCR techniques.

**Results:**

The results of culture showed that 52 (9.42%) and 28 (5.07%) of the total 552 milk and dairy samples were positive for presences of *Yersinia* species and *Y. enterocolitica*, respectively. Totally, 24 of 28 *Y. enterocolitica* isolates by culture were positive in PCR test (4.59%). Raw cow milk and traditional cheese had the highest prevalence of *Yersinia* species and *Y. enterocolitica, respectively*. There were no positive results for pasteurized cow milk, raw camel milk, commercial ice cream, commercial cheese, yoghurt, Doogh, butter and curd. *Yersinia* species and *Y. enterocolitica* had the highest prevalence in autumn (15.15% and 10.6%, respectively). Significant differences regarding P < 0.05 were observed between the presences of *Yersinia* species and *Y. enterocolitica* in various samples and seasons.

**Conclusions:**

Sanitation and pasteurization are the best ways to increase the microbial quality and particularly decrease the load of *Yersinia* species. The ability of *Yersinia* species to growth in Doogh, yoghurt, curd and butter is very low.

## 1. Background

*Yersinia enterocolitica* is a small rod-shaped Gram-negative coccobacillus psychrotrophic enterobacterium, isolated from a variety of environmental sources, foods, and human clinical samples (1-4). It is a causative organism in several outbreaks of gastroenteritis, in which foods were implicated (3, 5). It seems that foods with animal origins have the highest risk of gastrointestinal diseases caused by *Y. enterocolitica* in humans (1, 2, 6). Milk and dairy products are the most consumed foods with animal origins. In addition, several studies have reported the presence of *Y. enterocolitica* in milk and dairy products (1, 2, 7-10). *Y. enterocolitica* has a particular public health importance, because of its capability of growing in raw milk and viability at refrigeration temperatures for long time. Therefore, consumption of milk and dairy products has a higher chance of infection by *Y. enterocolitica* in humans. Consumption of raw milk and traditional dairy products is common in some areas of Iran; but, the most important issue about *Y. enterocolitica* is its surveillance in pasteurized milk and even commercial dairy products.

## 2. Objectives

The dairy-borne issue of *Y. enterocolitica* is essentially unknown in Iran. Therefore, the present study was carried out to investigate the prevalence of *Yersinia* species and *Y. enterocolitica* in raw and pasteurized milk as well as traditional and commercial dairy products in Isfahan province, Iran

## 3. Materials and Methods

### 3.1. Samples Collection

From January 2011 to January 2012, a total of 285 diary product samples including commercial cheese (n = 30), traditional cheese (n = 60), commercial ice cream (n = 15), traditional ice cream (n = 35), commercial yoghurt (n = 10), traditional yoghurt (n = 25), commercial Doogh (10), traditional Doogh (20), commercial butter (n = 15), traditional butter (n = 25), commercial curd (15) and traditional curd (35) were purchased from supermarkets and retailers in various parts of Isfahan, Iran. Totally, all of these samples were solid except for commercial and traditional Doogh. All dairy products were kept under refrigeration in plastic bags; information about dates of production and assigned shelf lives was not presented. Besides, 267 milk samples including raw cow milk (n = 80), pasteurized cow milk (n = 30), raw sheep milk (n = 60), raw goat milk (n = 60), and finally raw camel milk (n = 37) were purchased from the same locations. All milk samples showed normal physical characterizations and after collection, they were immediately transferred to the laboratory in a cooler with ice-packs. Samples were collected from various seasons of the year.

### 3.2. Identification of *Yersinia* Species 

Totally, 25 mL of liquid samples and 25 g of solid samples were aseptically added to 100 mL 0.01 M phosphate buffered saline (PBS, pH 7.6) in a sterile stomacher plastic bag and homogenized for 2 minutes. The homogenates were incubated at 25°C for 10 minutes. Different enrichment procedures were applied. One of the methods was adding 20 mL of the homogenate to 80 mL of trypticase soya broth (TSB) (Oxoid, UK) and enriching the mixture at 25°C for 24 hours. Another procedure was adding 20 mL of the homogenate to 80 mL of PBS and enriching the mixture at 4°C for 14 days (cold enrichment). The samples were treated with KOH (0.5% KOH in 0.5% saline) to suppress the background flora after enrichment. Sub-culturing on selective CIN (cefsulodin-irgasan-novobiocin) agar plates (*Yersinia *selective agar base, Oxoid UK) was applied according to the method of the FDA (11). 

One to the five susceptible colonies of typical “bull’s eye” appearance on the CIN agar plates, if available, were streaked onto tryptone soya agar (Oxoid, UK) plates to create a pure culture. All the isolates from pure cultures were examined for Gram staining, utilization of Simmon’s citrate, motility at 35°C and 27°C, Voges-Proskauer reaction at 35°C and 27°C, Kligler’s iron agar reaction, urease activity, indole production, triple sugar iron butt, slant, nitrate reduction, H_2_S, lysine decarboxylase, ornithine decarbozylase, oxidase, catalase, dextrose, lactose, sucrose, salicin, maltose, mannitol, rhamnose, arabinose, raffinose, inositoland sorbitol (11) (Table 1).

**Table 1.** Biochemical Characterization of *Y. enterocolitica *Isolated From Milk and Dairy Products ^a^

Substrate or Test	Reaction	Substrate or Test	Reaction
**Gram staining**	-	Lysine decarboxylase	-
**Utilization of Simmon’s citrate**	-	Ornithine decarbozylase	+
**Motility at 35°C**	-	Oxidase ^b^	-
**Motility at 27°C**	+	Catalase	+
**Voges-Proskauer at 35°C**	-	Dextrose	A
**Voges-Proskauer at 27°C**	+	Lactose	-
**Xylose**	A	Sucrose	A
**Urease activity**	+	Salicin	-
**Indole production**	+	Maltose	A (delayed)
**Triple sugar iron butt**	A	Mannitol	A
**Slant**	A	Rhamnose	-
**Nitrate reduction**	+	Arabinose	A
**H_2_S**	-	Raffinose	-
**Sorbitol**	A	Inositol	A (delayed)

^a^ +, Positive; -, Negative; A, Acid.

^b^ Taxo N discs (BBL).

### 3.3. DNA Extraction and Polymerase Chain Reaction

The bacterial strains which were cultivated on CIN medium and biochemically identified as *Y. enterocolitica,* were confirmed using the PCR method. Purification of DNA from bacterial colonies was achieved using a genomic DNA purification kit (Fermentas, Germany) according to the manufacturer’s instructions. To specifically amplify the *Y. enterocolitica* 16S rRNA gene, a set of primers, Y1 (5’-AATACCGCATAACGTCTTCG-3’) and Y2 (5’-CTTCTTCTGCGAGTAACGTC-3’), was used, resulting in a PCR product of 330 bp (12). The PCR products were stained with 1% solution of ethidium bromide and visualized under the UV light after electrophoresis on 1.5% agarose gel.

### 3.4. Statistical Analysis

Differences in the prevalence of *Yersinia* spp. and *Y. enterocolitica* in various milk and dairy products were analyzed using a chi-square test in SPSS for Windows (release 18.0 standard version, SPSS Inc., Chicago, Illinois). The differences were considered statistically significant when P < 0.05.

## 4. Results

Results of the present study confirmed the presence of *Yersinia* spp. and especially *Y. enterocolitica* in milk and dairy products. The culture method showed that from a total of 552 milk and dairy samples, 52 (9.42%) were positive for presences of *Yersinia* spp. (Table 2). After biochemical analysis of positive samples, it was recognized that from a total of 552 samples, 28 (5.07%) were positive for *Y. enterocolitica* (Table 2). On the other hand, from a total of 52 *Yersinia* spp. isolates, 28 (53.84%) were *Y. enterocolitica*. Our results showed that raw cow milk (16.25%) and traditional cheese (23.33%) had the highest prevalence of *Yersinia* spp. and *Y. enterocolitica* among milks and dairy products, respectively (Table 2). Results showed that there were no bacteria in pasteurized cow milk, raw camel milk, commercial cheese, commercial ice cream and both types of yoghurt, Doogh, butter and curd (Table 2). After PCR, it was recognized that from a total of 552 samples, 24 (4.34%) were positive for *Y. enterocolitica* 16 srRNA gene (Table 2).

Table 3 shows the distribution of *Yersinia* spp. and *Y. enterocolitica* in dairy samples in various seasons. Autumn had the highest (15.15% and 10.6%) while summer had the lowest (5.71% and 2.14%) prevalence of *Yersinia* spp. and *Y. enterocolitica*, respectively.

**Table 2.** Prevalence of *Yersinia* spp. and *Y. enterocolitica* in Milk and Dairy Products in Iran Using Culture and PCR Method ^a^

Type of Food	Number of Samples	Culture Method		PCR Method
		*Yersinia* spp.	*Y. enterocolitica*	*Y. enterocolitica*
**Raw cow milk**	80	13 (16.25) ^b^	8 (10) ^b^	6 (7.5) ^b^
**Pasteurized cow milk**	30	-	-	-
**Raw sheep milk**	60	5 (8.33)	3 (5)	3 (5)
**Raw goat milk**	60	2 (3.33)	1 (1.66)	1 (1.66)
**Raw camel milk**	37	-^b^	-^b^	-^b^
**Cheese**	90	14 (15.55)^c^	7 (7.77)^c^	6 (6.66)^c^
**Commercial cheese**	30	-^b^	-^b^	-^b^
**Traditional cheese^d^**	60	14 (23.33)	7 (11.66)	6 (10)
**Ice cream**	50	2 (4)	1 (2)	1 (2)
**Commercial ice cream**	15	-^b^	-^b^	-^b^
**Traditional ice cream**	35	2 (5.71)^b^	1 (2.85)^b^	1 (2.85)^b^
**Yogurt**	35	-	-	-
**Commercial yogurt**	10	-	-	-
**Traditional yogurt**	25	-	-	-
**Doogh^c^**	30	-	-	-
**Commercial Doogh**	10	-	-	-
**Traditional Doogh**	20	-	-	-
**Butter**	40	-	-	-
**Commercial butter**	15	-	-	-
**Traditional butter**	25	-	-	-
**curd ** ^**e**^	50	-	-	-
**Commercial curd**	15	-	-	-
**Traditional curd**	35	-	-	-
**Total**	552	52 (9.42)	28 (5.07)	24(4.34)

^a^ Data are presented as No. (%).

^b^ A significant difference about P < 0.05.

^c^ A dairy product prepared by beating unflavored yogurt until smooth, and then diluting with water to a consistency similar to whole milk; it is also called yogurt soda.

^d^ Made from raw sheep or cow milk.

^e^ A dairy product prepared by prolonged boiling yogurt.

**Table 3.** Distribution of *Yersinia* spp. and *Y. enterocolitica* in Dairy Samples During Various Seasons ^a^

Season	Number of Samples	Positive Samples for *Yersinia* spp. ^b^	Positive Samples for *Y. enterocolitica*
**Autumn**	132	20 (15.15) ^c^	14 (10.6) ^c^
**Winter**	140	11 (7.48)	4 (2.72)
**Spring**	133	13 (9.77)	7 (5.26)
**Summer**	147	8 (5.71) ^c^	3 (2.14) ^c^
**Total**	552	52 (9.42)	28 (5.07)

^a^ data are presented as No. (%).

^b^ These amounts were achieved from the culture method.

^c^ A significant difference about P < 0.05.

## 5. Discussion

Milk is raised as a complete food, especially for children and seniors. Its high values of proteins, minerals, fats and vitamins are undeniable. It is the primary source of nutrition for young mammals before they are able to digest other types of foods. In addition, milk has been transformed into various dairy products such as cheese, cream, butter, yogurt, kefir, Doogh, curd and ice cream. In a day, millions of people use milk and dairy products. Therefore, hygienic quality of milk and dairy products is very important; but sometimes it decreases, causing several infections and illness.

Foodborne diseases are among worldwide growing health problems, including a wide spectrum of illnesses caused by microbial, viral, parasitic or chemical contamination of food. Previous reports showed that among all foodborne pathogens such as *Y. enterocolitica*, *Clostridium botulinum*, *Campylobacter jejuni*, *Escherichia coli* O157:H7, *Listeria monocytogenes*, *Salmonella* spp., *Shigella* spp. and *Bacillus cereus*, the first one is the most common foodborne bacterium in foods with animal origins (6, 13, 14). As far as we know, several investigations have reported outbreaks of foodborne infections caused by *Y. enterocolitica*, in which contaminated milk was incriminated (15, 16).

To our knowledge, such surprisingly high percentage of positive results (9.42%) had never been reported in Iran. Only one study has been conducted on detection of *Y. enterocolitica* in milk in Iran. This study showed that five samples (1.6%) of raw milk, but no pasteurized milk samples, were positive for *Y. enterocolitica *(1), which was lower than our results. Another study in Iran showed that 15.8% of meat and chicken samples were contaminated with *Y. enterocolitica *(17). Our study showed significant differences (P < 0.05) in the presences of *Yersinia* spp. and *Y. enterocolitica, *in comparison of raw cow milk with pasteurized cow milk and raw camel milk. Furthermore, there were significant differences in the presences of *Yersinia* spp. and *Y. enterocolitica, *comparing traditional cheese with commercial cheese and traditional ice cream with commercial ice cream (P < 0.05). It seemed that there was no significant difference in confidence level of 95% between the ability of culture with PCR technique for detection of *Yersinia* spp. and *Y. enterocolitica*.

In a study in Morocco, *Yersinia* spp. was recovered from 11 of 30 raw milks (36.6%), 1 of 20 pasteurized milks (5%), 15 of 63 traditional fermented milks (23.8%), 7 of 94 cheeses (7.44%), and 1 of 20 cream samples (5%), and the overall incidence of *Y. enterocolitica* in milk and dairy products was 6.6% (18), which was slightly higher than our results (5.07%). Another study showed that the prevalence of *Y. enterocolitica* was 24.1% in raw buffalo milk; however, no isolation could be made from the pasteurized milk samples (19) and this was in agreement with our research. Our study was the first prevalence reports of *Yersinia* spp. and *Y. enterocolitica* in camel milk, Doogh, curd, butter and yoghurt samples in Iran. In our study, there were no positive results for presences of *Yersinia* spp. and *Y. enterocolitica* in the above samples. Therefore, there was no possibility for the presence of *Yersinia* spp. and *Y. enterocolitica* in the pasteurized milk and camel milk. In despite of these two types of samples, other types of dairy products can be reservoir for Yersinia spp.

Curd is a dairy product prepared by prolonged boiling of yogurt. Doogh is a dairy product prepared by beating unflavored yogurt until smooth, and then diluting with water to a consistency similar to whole milk; it is also called yogurt soda. Different pH values have the golden role in the prevalence of *Y. enterocolitica* in different samples. Bhaduri et al. (20) reported no development of *Y. enterocolitica* at pH = 4.5 and low temperatures (5°C to 19°C). Brackett (21) found that this bacterium remained viable at pH = 4 for at least 21 days at 5°C. The *Y. enterocolitica *isolated from milk and dairy samples of our study, was recovered from samples with pH of 4.8-5 at 4°C. Probably, curd, Doogh and yoghurt, studied in our research, had high activated water. Therefore, these samples had high acidity and there were no possibility for their bacterial contamination.

Old studies conducted on raw milk in Alsace and France showed that out of 75 tested samples, 61 (81.4%) were contaminated with *Yersinia* spp. (22). An Irish study showed that 279 of 589 dairy samples were contaminated with *Yersinia* spp., 59% of which were *Y. enterocolitica *(23), which was higher than our results (53.84%). A Turkish study performed on 211 raw milk samples, revealed 33 pathogenic microorganisms, among which eight were *Y. enterocolitica *(24). A study in Pennsylvania showed a lower amount of bulk tank milk contamination with *Y. enterocolitica* (1.2%) (10).

Our results showed that traditional ice creams were one of the main sources of *Y. enterocolitica* (2.85% prevalence rate). In a previous study, *Y. enterocolitica *was isolated from samples of ingredients used in the production of ice cream, such as cream, egg, and pasteurized milk (16). Furthermore, several studies have reported isolation of *Y. enterocolitica *from ice cream (25, 26).

A previous study showed that the occurrence of *Y. enterocolitica *was slightly higher in processed milk (73.8% positive) than bulk tank milk (64.7%) (22) and this was in contrast with our results. A study in USA (14) showed that from a total of 292 bulk tank milk samples, 4.1%, 12.1%, 15.1% and 8.9% of samples were positive for *L. monocytogenes*, *C. jejuni*, *Y. enterocolitica* and *salmonella*, respectively. Therefore, *Y. enterocolitica* had the highest prevalence among milk-borne pathogens. In a study in Dakota, *C. jejuni*, shiga-toxin producing *E. coli*, *L. monocytogenes*, *Salmonella* spp. and *Y. enterocolitica* were detected in 9.2%, 3.8%, 4.6%, 6.1%, and 6.1% of bulk tank milk samples, respectively (10).

In a study in Turkey, from a total of 100 white cheese samples purchased from retailers, supermarkets and factories, 8 (8%) were positive for presence of *Y. enterocolitica* using the culture method (27), which was in agreement with our study. Another study in Turkey showed that from a total of 14 and 55 *Yersinia* spp. isolates, respectively isolated from raw milk and traditional cheese with pH of 4-5, 7.1% were *Y. intermedia,*47.3% *Y. enterocolitica*, 31% *Y. fredriksen*, 7.2% *Y. intermedia*, 12.7% *Y. Christensen* and finally 1.8% were other species of *Yersinia *(28).

Our results showed that the prevalence of *Yersinia* spp. and *Y. enterocolitica* in milk and dairy products had a seasonal pattern. These bacteria had the highest prevalence in autumn and the lowest in winter. Statistical analysis showed significant differences in the presence of *Yersinia* spp. and *Y. enterocolitica *between milk and dairy samples of autumn and summer (P < 0.05). Another study showed that the incidence of *Y. enterocolitica* in milk and dairy products were much higher (25-50%) during winter compared with summer (0-17%) (19). Seasonal prevalence of this bacterium was previously reported (29-32), in all of which the prevalence of *Y. enterocolitica* was higher in warmer seasons.

Rare cases of mastitis have been associated with *Yersinia* spp. in Israel (33). More commonly, risk factors for contamination of raw milk are likely to be those associated with poor hygiene of milking and fecal contamination of the teat ends prior to milking cup attachment. The differences between findings of various authors and those of this study might be due to several factors such as method of sampling, number of analyzed samples, method of analyzing, sources of samples, season, and geographical location. These factors may cause an increase or decrease in the incidence of *Yersinia* spp. infection. Neither vaccines for prevention of yersiniosis in animals nor any routinely available tests for subclinical infections are currently available. Control of this pathogen relies on effective hygiene management of the farm, especially the milking practice.

 Our results showed that using raw milk without pasteurization, milking with unsanitary methods, and using traditional dairy products produced in unsanitary conditions and probably from unpasteurized milk, are the main resources for growth, proliferation and survival of *Y. enterocolitica*. These factors cause several disorders for human. Therefore, improving the methods of milking, monthly checking of the milking halls to detect *Y. enterocolitica* especially in the animal feces, fumigating the milking halls frequently, inspecting the hygiene during milking, boiling the milk, using pasteurized and even sterilized milk for dairy products, keeping dairy products in cool and dry places away from the sunlight, and finally preventing from contamination of dairy products with extrinsic factors such as insects and dust, are the best ways to prevent *Y. enterocolitica* infections.

## References

[1] Soltan-Dallal MM, Tabarraie A, MoezArdalan K (2004). Comparison of four methods for isolation of Yersinia enterocolitica from raw and pasteurized milk from northern Iran.. Int J Food Microbiol..

[2] Myers KM, Gaba J, Al-Khaldi SF (2006). Molecular identification of Yersinia enterocolitica isolated from pasteurized whole milk using DNA microarray chip hybridization.. Mol Cell Probes..

[3] Lambertz ST, Nilsson C, Hallanvuo S, Lindblad M (2008). Real-time PCR method for detection of pathogenic Yersinia enterocolitica in food.. Appl Environ Microbiol..

[4] Thoerner P, Bin Kingombe CI, Bogli-Stuber K, Bissig-Choisat B, Wassenaar TM, Frey J (2003). PCR detection of virulence genes in Yersinia enterocolitica and Yersinia pseudotuberculosis and investigation of virulence gene distribution.. Appl Environ Microbiol..

[5] Grahek-Ogden D, Schimmer B, Cudjoe KS, Nygard K, Kapperud G (2007). Outbreak of Yersinia enterocolitica serogroup O:9 infection and processed pork, Norway.. Emerg Infect Dis..

[6] Arnold T, Neubauer H, Nikolaou K, Roesler U, Hensel A (2004). Identification of Yersinia enterocolitica in minced meat: a comparative analysis of API 20E, Yersinia identification kit and a 16S rRNA-based PCR method.. J Vet Med B Infect Dis Vet Public Health..

[7] Hayaloglu AA, Guven M, Fox PF (2002). Microbiological, biochemical and technological properties of Turkish White cheese ‘Beyaz Peynir’.. Int Dairy J..

[8] Akin N, Aydemir S, Kocak C, Yildiz MA (2003). Changes of free fatty acid contents and sensory properties of white pickled cheese during ripening.. Food Chem..

[9] Guven A, Sezer C, Aydin BD, Oral NB, Vatansever L (2010). Incidence and Pathogenicity of Yersinia enterocolitica Isolates from Foods in Turkey.. Kafkas Univ Vet Fak Derg..

[10] Jayarao BM, Donaldson SC, Straley BA, Sawant AA, Hegde NV, Brown JL (2006). A survey of foodborne pathogens in bulk tank milk and raw milk consumption among farm families in pennsylvania.. J Dairy Sci..

[11] Weagant SD, Feng P, Food and Drug Administrator.. (2007). Yersinia enterocolitica. Bacteriological Analytical Manual Online..

[12] Wannet WJ, Reessink M, Brunings HA, Maas HM (2001). Detection of pathogenic Yersinia enterocolitica by a rapid and sensitive duplex PCR assay.. J Clin Microbiol..

[13] World Health Organization (2007). Food Safety and Food-borne Illness. Fact Sheet No. 237..

[14] Draughon FA, Davidson PM, Oliver SP (1992). Prevalence of Listeria monocytogenes, Campylobacter jejuni, Yersinia enterocolitica, and Salmonella in bulk tank milk: risk factors and risk of human exposure.. J Food Protect..

[15] Black RE, Jackson RJ, Tsai T, Medvesky M, Shayegani M, Feeley JC (1978). Epidemic Yersinia enterocolitica infection due to contaminated chocolate milk.. N Engl J Med..

[16] Tacket CO, Narain JP, Sattin R, Lofgren JP, Konigsberg C, Jr., Rendtorff RC (1984). A multistate outbreak of infections caused by Yersinia enterocolitica transmitted by pasteurized milk.. JAMA..

[17] Soltan-Dallal MM, Zali MR, Avadisians S, Bakhtiari R (2011). Incidence and antibiotic susceptibilities of Yersinia enterocolitica and other Yersinia species recovered from meat and chicken in Tehran, Iran.. Afr J Microbiol Res..

[18] Hamama A, el Marrakchi A, el Othmani F (1992). Occurrence of Yersinia enterocolitica in milk and dairy products in Morocco.. Int J Food Microbiol..

[19] Toora S, Singh G, Tiwari RP, Singh G (1989). Drug resistance and lecithinase activity of Yersinia enterocolitica isolated from buffalo milk.. Int J Food Microbiol..

[20] Bhaduri S, Buchanan RL, Phillips JG (1995). Expanded response surface model for predicting the effects of temperatures, pH, sodium chloride contents and sodium nitrite concentrations on the growth rate of Yersinia enterocolitica.. J Appl Bacteriol..

[21] Brackett RE (1986). Growth and survival of Yersinia enterocolitica at acidic pH.. Int J Food Microbiol..

[22] Vidon DJ, Delmas CL (1981). Incidence of Yersinia enterocolitica in raw milk in eastern France.. Appl Environ Microbiol..

[23] Rea MC, Cogan TM, Tobin S (1992). Incidence of pathogenic bacteria in raw milk in Ireland.. J Appl Bacteriol..

[24] Uraz G, Yucel N (1999). The isolation of certain pathogen microorganisms from raw milk.. Cent Eur J Public Health..

[25] Schiemann DA (1987). Yersinia enterocolitica in milk and dairy products.. J Dairy Sci..

[26] Sarigiannidou D, Koidis P, Batzios C, Mantis A (1997). Behaviour of Yersinia enterocolitica in Mizithra cheese.. Arch Lebensmittelhygiene..

[27] Yentur G, Kocaman NB, Er B, Oktem AB (2008). Occurrence of yersinia enterocolita in turkish white cheese consumed In karabuk region-turkey.. Turk J Pharm Sci..

[28] Yucel N, Ulusoy H (2006). A Turkey survey of hygiene indicator bacteria and Yersinia enterocolitica in raw milk and cheese samples.. Food Control..

[29] Weber A, Knapp W (1981). [Seasonal isolation of Yersinia enterocolitica and Yersinia pseudotuberculosis from tonsils of healthy slaughter pigs (author's transl)].. Zentralbl Bakteriol Mikrobiol Hyg A..

[30] Weber A, Knapp W (1981). Nachweis von Yersinia enterocolitica und Yersinia pseudotuberculosis in Kotproben gesunder Schlachtschweine in Abhängigkeit von der Jahreszeit.. Zentralblatt für Veterinärmedizin Reihe B..

[31] Fredriksson-Ahomaa M, Korte T, Korkeala H (2001). Transmission of Yersinia enterocolitica 4/O:3 to pets via contaminated pork.. Lett Appl Microbiol..

[32] Fredriksson-Ahomaa M, Korkeala H (2003). Low occurrence of pathogenic Yersinia enterocolitica in clinical, food, and environmental samples: a methodological problem.. Clin Microbiol Rev..

[33] Shwimmer A, Freed M, Blum S, Khatib N, Weissblit L, Friedman S (2007). Mastitis caused by Yersinia pseudotuberculosis in Israeli dairy cattle and public health implications.. Zoonoses Public Health..

